# Psychometric properties of the Chinese version of the Family Questionnaire among the caregivers of people with schizophrenia

**DOI:** 10.3389/fpubh.2023.1200130

**Published:** 2023-07-14

**Authors:** Yanan Peng, Enhe Xiong, Yan Li, Lanjun Song, Juzhe Xi

**Affiliations:** ^1^Shanghai Key Laboratory of Mental Health and Psychological Crisis, Affiliated Mental Health Center (ECNU), Positive Education China Academy (PECA) of Han-Jing Institute for Studies in Classics, Juzhe Xi's Master Workroom of Shanghai School Mental Health Service, School of Psychology and Cognitive Science, East China Normal University, Shanghai, China; ^2^Shanghai Changning Mental Health Center, Shanghai, China

**Keywords:** caregivers, expressed emotion, psychometrics, schizophrenia, validation study

## Abstract

**Introduction:**

Expressed emotion refers to relatives' attitudes and emotional behaviors toward mentally ill family members. It is a robust predictor of patients' illness outcomes and caregivers' wellbeing in a wide range of mental disorders. However, expressed emotion has not been fully explored in the Chinese context. One reason is the lack of reliable and cost-effective measurements. A reliable, valid, and user-friendly instrument is needed to support the research and clinical practice based on expressed emotion in China. This study aimed to translate, adapt, and examine the psychometric properties (factorial structure, measurement invariance, internal consistency reliability, and concurrent validity) of a Chinese version of the Family Questionnaire.

**Methods:**

A total of 248 caregivers participated in the study. A translation and back-translation procedure was applied to translate the Family Questionnaire into Chinese. We compared two models to examine the factor structure of the questionnaire by performing confirmatory factor analysis. We also conducted measurement invariance analysis to test whether the factor structure of the tool is invariant across male and female groups. Reliability was evaluated with Cronbach's α. The concurrent validity was examined by testing the predictivity of the expressed emotion on relevant outcomes with path analysis. We used the STROBE checklist to report.

**Results:**

The item-total correlation coefficients of the scale ranged from 0.375 to 0.752. The confirmatory factor analysis indicated that the Chinese version of the Family Questionnaire displays the original two-factor structure (emotional overinvolvement and criticism; *X*^2^ = 335.50, *df* = 169, *X*^2^/*df* = 1.985, RMSEA = 0.063, SRMR = 0.058, CFI = 0.913, and TLI = 0.902). In addition, the two-factor structure was invariant across the male and female groups. The two subscales showed excellent internal consistency, with Cronbach's alpha of 0.92 for both emotional overinvolvement and criticism. The concurrent validity of the Chinese version was supported by the good predictivity of the two subscales to care burden, family function, and quality of life. All path coefficients were significant, and the absolute values of path coefficients ranged from 0.23 to 0.72.

**Conclusion:**

The Chinese version of the Family Questionnaire is a valid and reliable measurement of expressed emotion in the Chinese context.

## 1. Introduction

In recent decades, the care for schizophrenia has shifted from institution to community-based, with the belief that community-based care is better for patients' recovery (1). Most schizophrenia outpatients are cared for by their relatives, such as their parents, spouses, and siblings (2). Under this setting, the family environment and the interaction between caregivers and patients significantly impact patients' recovery. As an important family-level stress factor, expressed emotion refers to relatives' attitudes and emotional behaviors toward mentally ill family members. Expressed emotion was conceptualized as an environmental stressor that would increase the probability of psychosis development among people at high genetic risk for mental disorders (3–5). It is a robust predictor of patients' illness outcomes in various mental disorders, including schizophrenia, mood disorders, eating disorders, and dementia (6–11). Over the past decades, meta-analyses and review articles have shed light on its significant impact on mental health disorders (6, 10, 12–15). Despite the substantial empirical research in developed countries and areas, we still need more exploration of expressed emotion from different cultural contexts to expand our understanding of this construct.

Brown (16) first developed the construct of expressed emotion in the 1960's through his clinical practice on people with schizophrenia. Based on Brown and Rutter's work (17), expressed emotion has been studied as an index of family stress to predict symptom relapse in a wide range of mental disorders during the past decades (4, 6, 10–13, 18, 19). In the beginning, expressed emotion comprises five components (20): (1) criticism, which refers to family members' blame or disapproval of the patient's behavior; (2) hostility, which refers to rejection or dislike toward the patient; (3) emotional overinvolvement, which refers to relatives' extravagant/exaggerated emotional responses (e.g., anxiety and worry), and over-protection toward patients; (4) warmth, which reflects empathy and understanding toward the patients; (5) positive remarks, which refer to an appreciation of the patients. Subsequent research found that the first three components (i.e., criticism, hostility, and emotional overinvolvement) showed better predictivity to relapse (5, 6, 21, 22), so the key elements of expressed emotion are considered as criticism (CC), hostility, and emotional overinvolvement (EOI). Given that hostility is associated with high levels of criticism, the ratings of emotional overinvolvement and criticism are most used to classify caregivers into high or low levels of expressed emotion (23–25).

A variety of instruments were developed to measure expressed emotion. The first standardized measurement is the Camberwell Family Inventory (CFI) (26). CFI administration consists of two parts that require trained personnel: interviewing and coding. Interviewing usually takes 1–2 h, and coding takes 2–3 h. The time-consuming administration and coding and the required training of raters limit the use of CFI. Thus, researchers have developed alternative measures with shorter procedures. One alternative is the Five-Minute Speech Scale [FMSS; (27)]. FMSS reduced the administration time by fixing the interview to 5 min and removing the rating of hostility and warmth. However, the FMSS tends to under-identify high-expressed emotion relatives and inflate Type II errors in exploring the relationship between FMSS-rated expressed emotion and any given outcomes (28). Other alternative measures are self-report questionnaires, which are time- and cost-effective ways to measure expressed emotion. Self-report questionnaires also dispense with the dichotomous high/low rating of expressed emotion that has previously been criticized (29).

There are numerous self-report questionnaires used by researchers to measure EE. However, only a few were developed based on the EE construct and validated against the CFI. These self-report questionnaires are the Level of Expressed Emotion Scale (LEE), Family Attitude Scale (FAS), Perceived Criticism Measure (PCM), and Family Questionnaire (FQ). The LEE (30) is a 60-item scale with four subscales. FAS (31) is a unidimensional self-report measure with 30 items. The PCM (32) has only four items with a Likert scale of 10 points to measure criticism. These three scales are all valid measurements and have been used in EE research. In addition to their advantages, these questionnaires also have disadvantages. LEE contains multiple dimensions but is relatively lengthy. FAS is short but cannot measure different elements of EE separately. It is extremely fast and easy to assess PCM, but it only offers information about criticism. Thus, a short valid scale that can assess the main elements of EE to probe high-EE attitudes and is easier to administer and less time-consuming is required. These conditions can be satisfied by the FQ.

The FQ is a cost-effective and research-applicable self-report tool developed by Wiedemann et al. (25). First, the researchers generated 130 items from three different sources: common statements made by relatives of people with schizophrenia and behaviors of such relatives listed by experienced clinicians, expressed emotion-related concepts, and existing questionnaires. Second, based on the theoretical model developed by Vaughn and Leff (26, 33, 34), items were generated for four areas: “intrusiveness,” “emotional response,” “attribution of illness,” and “coping skills.” Third, the item pool was evaluated by a team of expressed emotion experts comprised of experienced clinicians familiar with people with schizophrenia, patients' relatives, and the expressed emotion literature. Finally, after a series of psychometric evaluations, 20 items were selected from the 130 preliminary items to measure expressed emotion's two critical elements (criticism and emotional overinvolvement). The FQ is equivalent to the FMSS in terms of validity but is easier to administer and less time-consuming than the CFI or the FMSS (25). In addition, it is suitable for repeated administration because no training is required before use, and the time needed for administration is short. The original version of the FQ showed good validity and was used in many empirical studies. The FQ also showed good psychometrics in other cultural contexts, including Italian (35), Greek (36), Brazilian Portuguese (37), and Spanish (38).

Although expressed emotion has been deeply researched in Western countries, it has not aroused much attention in China. The first batch of Chinese expressed emotion studies was a series of studies (39–43) conducted by Phillips and his cooperators in China around 2000. This team adapted CFI for use in China and evaluated the expressed emotion level of relatives of people with schizophrenia in Beijing. They investigated the relationship between some social demographic factors and expressed emotion, finding that the manifestation of expressed emotion varied in relatives with different sex, roles, educational level, and the length of time contact with the patient (39–42). They also found that relatives' stigma was positively associated with the high level of expressed emotion (41) and expressed emotion mediated the effect of controllable attributions on relapse in the Chinese sample (43). In the following decades, research on expressed emotion in China did not increase much. Several recent studies (44, 45) with samples from Hong Kong and China continue to support that high EE predicts rehospitalization of schizophrenic patients and caregivers' care burden and poor wellbeing. The lack of training opportunities for CFI use, time constraints in Chinese clinical practice, and the time-consuming administration of CFI may be part of the reasons for the lack of research about expressed emotion in China. Thus, time- and cost-effective measurements of expressed emotion that meet local clinical needs would be helpful to research and clinical practice about expressed emotion in China. The FQ could be an appropriate tool to use in China.

The existing valid Chinese version of self-reported expressed emotion measurements are the Level of Expressed Emotion Scale [LEE; (46–50)] and Family Attitude Scale [FAS; (51)]. Using samples of Hong Kong people with schizophrenia and other mental health disorders, Chien et al. (46–48) refined the LEE (Patient Version) into a 52-item Chinese version. Ng and Sun and Ng et al. (49, 50) further developed a 12-item Concise Chinese Level of Expressed Emotion Scale (CCLEES) after taking into account the limitations of Chien's 52-item Chinese LEE. According to Ng and Sun and Ng et al. (49, 50), the 12-item CCLEES is over four times shorter than Chien's but still accurate in assessing three core elements of EE. Chien's 52-item Chinese LEE and Ng's 12-item CCLEES are both measures for patients to report the level of EE they perceived from relatives. Using caregivers' self-reported data, Yu et al. (51) translated and validated a Chinese version of the FAS. However, it was based on a sample of caregivers for people with dementia. The Chinese version of FAS has not been tested on caregivers of people with schizophrenia or other mental health disorders.

While the existing Chinese self-report scales are valid, we believe using the FQ to evaluate EE in China has several advantages. First, it takes little time to administer and evaluate, and it does not require any training to use. Second, the FQ is concise while measuring the two critical elements of EE (i.e., EOI and criticism). The Chinese version of LEE with 52 items is relatively long for clinical and research settings. The FAS focuses on criticism and hostility without much information about EOI. In addition, Phillips and Xiong (39) noticed that the construct of criticism and emotional overinvolvement defined in CFI were more relevant dimensions in the Chinese context than the other three. The empirical studies using the CFI also showed that criticism and emotional overinvolvement were more common in Chinese relatives of people with schizophrenia (40, 42). Third, given the evidence available (35–38), the FQ tends to show a stable factor structure across cultural contexts. This facilitates cross-cultural EE comparisons. Instead, the structure of LEE seems unstable, and the CCLEES was less used in other cultural contexts. In sum, an adapted Chinese version of the FQ (C-FQ) would benefit research and clinical practice about expressed emotion in China.

In this study, we aimed to translate, adapt, and examine the psychometric properties of the C-FQ in a Chinese sample of caregivers of people with schizophrenia. Specifically, we would verify its (1) two-factor structure via confirmatory factor analysis; (2) internal consistency reliability; (3) concurrent validity with constructs related to expressed emotion (family function, care burden, and quality of life); and (4) measurement invariance across sex.

## 2. Methods

### 2.1. Translation of the C-FQ

Translation and back-translation procedure was applied to translate the FQ into Chinese. To start with, two bilingual psychologists who are both native speakers of Chinese and advanced speakers of English independently translated the questionnaire into Chinese (forward translation). Then, a reconciliation meeting was conducted to develop a consensus version (reconciliated Chinese version) with the help of a third reviewer. After that, two psychologists who were blind to the original version translated the reconciliated Chinese version back into English (backward translation). A third reviewer compared the backward translation and the original English version and decided that no significant discrepancies existed between the two versions, thus formulating the revised C-FQ. The English and Chinese versions of the items are listed in the Supplementary material.

### 2.2. Procedure and participants

We recruited relatives of people with schizophrenia from four communities in a district of Shanghai. They are primary caregivers of patients in a public mental health hospital. The participants' inclusion criteria were as follows: participants who (1) were aged 18 years or above; (2) were primary caregivers of the patient; (3) were without a diagnosis of mental health disorder; and (4) were able to read and write Chinese. The sample size estimation was guided by a rule of thumb with at least 10 respondents for each item in factor analysis. Considering the 20 items in the Family Questionnaire, the minimum sample size required was 200 participants.

We collected data for this study between September and October 2019. Data were collected when community doctors from the mental health hospital visited the family for a routine check. First, the doctor would briefly introduce the study to the caregiver, and a research assistant would expand on details about the purpose, procedure, incentive, data confidentiality, and participants' rights. If interested and willing to participate, the caregiver would sign the informed consent and complete a battery of questions.

Finally, 248 caregivers participated in this study. All of them were Chinese speakers. Table 1 summarizes the demographic of the caregivers and patients they care for.

**Table 1:** Demographic information of caregivers and the patients they take care of (*N* = 248).

**Caregivers**	**% (*n*)/*M* (*SD*)**
Age	65.19 (12.41)
**Gender**	
Male	52.42 % (130)
Female	47.58% (118)
**Educational level**	
Primary school	11.29% (28)
Junior high school	42.74% (106)
High school	34.27% (85)
Undergraduate	10.89% (27)
Did not report	0.81% (2)
**Roles**	
Father	35.08% (87)
Mother	36.69% (91)
Spouse	14.92% (37)
Siblings	9.27% (23)
Others	4.03% (10)
**Living with the patients**	
Yes	79.84% (198)
No	19.76% (49)
Did not report	0.40% (1)
Contact time with the patients per week (h)	83.67 (59.29)
**Household monthly per capita income (CNY)**	
Under 3,000	18.55% (46)
3,001–5,000	63.71% (158)
5,001–10,000	15.32 % (38)
More than 10,000	1.61% (4)
Did not report	0.81% (2)
Illness duration of patients	20.17 (8.75)
**Taking medicine (patients)**	
Yes	90.32% (224)
No	9.68% (24)

### 2.3. Ethics approval and consent to participate

The study was approved by the East China Normal University Committee on Human Research Protection (IRB No: HR 012-2019). Written informed consent was received from all participants. All study details were disclosed to the participants. Participants were free to withdraw from the study at any time.

### 2.4. Instruments

#### 2.4.1. Expressed emotion

Expressed emotion of caregivers was measured by the Family Questionnaire (25). This questionnaire was evaluated as a reliable psychometric tool applied to different cultures (35–38). It is composed of two subscales: emotional overinvolvement (10 items) and criticism (10 items). Items were rated on a 4-point Likert scale from 1 (strongly disagree) to 4 (strongly agree), and a higher score indicated a higher level of expressed emotion. The Cronbach's alpha for emotional overinvolvement and criticism was both 0.92 in the current study.

#### 2.4.2. Family function

The Chinese version of the Family Assessment Device (52) was used to evaluate family functions. The scale consists of seven subscales: problem-solving, communication, roles, affective responsiveness, affective involvement, behavior control, and general functioning. Items were rated on a 4-point Likert scale from 1 (*strongly disagree*) to 4 (*strongly agree*). Lower scores indicate healthier family functioning. A review (53) summarized the performance of the Chinese version of the Family Assessment Device and found that it has shown good reliability and validity in Chinese participants since its validation. This scale also achieved high reliability (0.79–0.92) in recent studies using Chinese adult samples (54, 55). In this study, Cronbach's alpha for this device was 0.88.

#### 2.4.3. Quality of life

Quality of life was measured with the Chinese version of the WHOQOL-BREF (56). Four subscales make up the WHOQOL-BREF, including the physical domain, psychological domain, social relationships, and environmental domain. The number of questions was cut down in this study in case participants get overwhelmed with too many items (57). Finally, items of the environmental domain were excluded from this study, for this domain is more affected by the public environment than the family system. All items were rated on a 5-point Likert scale from 1 to 5. Higher scores indicate a higher level of quality of life. In comparison and review studies (58, 59), WHOQOL-BREF has been shown to be a sound, cross-culturally valid assessment of QOL in various countries (including China). Previous psychometric studies (56, 60, 61) have also indicated its high reliability (Cronbach's α > 0.88) in the Chinese adult population. In this study, Cronbach's alpha of the scale was 0.90.

#### 2.4.4. Caregiver burden

The burden of caregivers was measured by the Chinese version of the Zarit Burden Interview (62). There are 22 items, rating on a 5-point Likert scale from 0 (never) to 4 (always). Higher scores indicate a higher level of burden. The Chinese version of the Zarit Burden Interview has demonstrated high internal consistency (Cronbach's α > 0.87) in psychometric assessment studies based on samples of caregivers of older adults, inpatients, and people with schizophrenia (62–64). The Cronbach's alpha of this scale was 0.96 in this study.

### 2.5. Data analyses

The whole process of statistical analysis was conducted by MPLUS 7.4 statistical program (65) except the association between C-FQ and socio-demographic variables, and clinical characteristics were estimated by SPSS Statistics 25 software. Full information maximum likelihood (FIML) was used to handle missing data, and Maximum Likelihood Robust (MLR) was used in the analyses. Preliminary analyses (skewness, kurtosis, and item-total correlation) were conducted to test the normality of every item in the FQ (66) and to exclude poor-fitting items.

Later, confirmatory analyses were conducted to test the validity of the Chinese version of FQ (C-FQ). The fit of the model was tested with several indices, including χ^2^, the comparative fit index [CFI; (67)], Tucker and Lewis index [TLI; (68)], root mean square error approximation [RMSEA; (69)], and the standardized root mean square residual [SRMR; (70)]. A model can be considered satisfactory with the CFI and the TLI both over 0.90 (71) and the values of the RMSEA and the SRMS < 0.08 (72). We also compared two models to examine whether the structure of C-FQ was similar to the original one. Specifically, one was a single-factor model where all items load onto the general factor of expressed emotion (Model 1). The other was a model with two intercorrelated factors (critical comments and emotional overinvolvement) as in previous research (25, 36, 38) (Model 2). In addition, we used Satorra-Bentler scaled chi-square difference test (73) to compare the fitness of the two models.

Then, the measurement invariance of factor structure (configural invariance), factor loadings (metric invariance), and intercepts (scalar invariance) across gender were examined using the whole sample. ΔCFI and ΔTLI were used to investigate measurement invariance. Comparing models where loadings and thresholds were held equal vs. free to vary, a reduction in CFI (ΔCFI) and TLI (ΔCFI) of < 0.01 suggests that the model is scalar and metric invariant (74, 75). Cronbach's alpha was calculated to evaluate the internal consistency reliability of the scale. Moreover, concurrent validity was evaluated by path analysis, where all variables were specified as explicit ones. Concurrent validity is usually determined by correlation coefficients between criterion and target scores (76). Based on the most common guidelines, a strong correlation is defined as *r* between 0.75 and 1, while a moderate correlation is defined as *r* between 0.30 and 0.70 (77). The non-parametric test was used to examine the association of C-FQ with socio-demographic variables and clinical characteristics since the Kolmogorov–Smirnov test revealed a non-normal distribution of C-FQ. Specifically, univariate associations between C-FQ and categorical variables were tested by Mann–Whitney and Kruskal–Wallis tests. The strength of the association between C-FQ and continuous variables was estimated via Spearman's rho correlation coefficient.

## 3. Results

### 3.1. Preliminary analyses

In preliminary analyses, item 17 presented a slightly non-normal distribution, with a kurtosis over 2 (66, 78). The item-total correlations for all items present acceptable values, so no item was deleted. Given the non-normal distribution of item 17, further data analyses were conducted using the MLR estimator (66). Table 2 shows all C-FQ items' mean, standard deviation, skewness, kurtosis, and item-total correlations.

**Table 2:** Mean, standard deviation, skewness, kurtosis, and item-total correlations of all C-FQ items.

	***M***	***SD***	**Skewness**	**Kurtosis**	**Item-total correlation**
Item 1	2.46	0.385	−0.388	−0.410	0.636
Item 2	2.36	0.417	−0.065	−0.298	0.661
Item 3	2.78	0.455	−0.647	0.731	0.691
Item 4	2.47	0.426	−0.315	−0.293	0.752
Item 5	2.55	0.513	−0.349	−0.169	0.745
Item 6	2.57	0.487	−0.245	−0.151	0.723
Item 7	2.29	0.466	0.017	−0.273	0.717
Item 8	2.44	0.464	−0.049	−0.247	0.651
Item 9	2.47	0.460	−0.046	−0.233	0.597
Item 10	2.31	0.457	0.331	0.106	0.545
Item 11	2.53	0.436	−0.393	−0.163	0.703
Item 12	2.63	0.468	−0.343	0.001	0.736
Item 13	2.68	0.451	−0.411	0.189	0.705
Item 14	2.14	0.402	0.069	−0.140	0.542
Item 15	2.42	0.504	−0.121	−0.323	0.660
Item 16	2.40	0.419	−0.242	−0.395	0.663
Item 17	2.88	0.342	−0.828	2.021	0.375
Item 18	2.52	0.395	−0.256	−0.250	0.735
Item 19	2.58	0.431	−0.493	−0.006	0.742
Item 20	2.26	0.313	0.266	0.040	0.580

### 3.2. Confirmatory factor analyses

**Figure 1:**
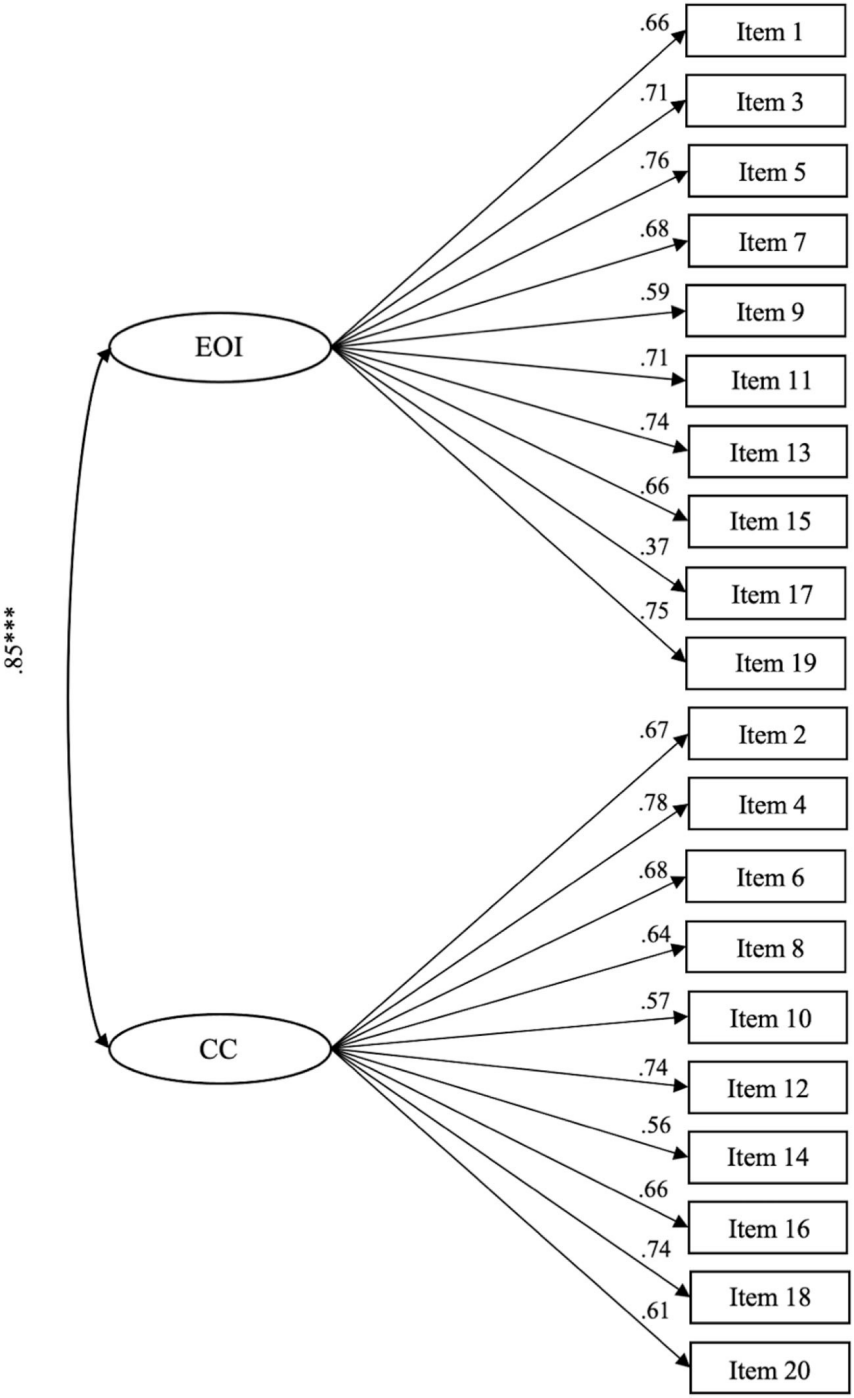
Standardized solution of the factor structure of the C-FQ. CC, criticism; EOI, emotional overinvolvement; the R-SQUARE values of all items vary from a minimum of 0.135 to a maximum of 0.603; ***p < 0.001.

As stated above, the confirmatory factor analysis was conducted using the MLR estimator. Reported in Table 3 are the fit indices of two models, which manifests that the single-factor model (Model 1) was not acceptable. In contrast, the two-factor model (Model 2) was a preferable structure, with satisfactory fit indices, all factorial loadings being significant (*p* < 0.001), and a significant improvement in model fit compared to Model 1. Moreover, the dimension of emotional overinvolvement and criticism is positively correlated with each other (*p* < 0.001; see Figure 1).

**Table 3:** Model fit indices for confirmatory factor analyses.

	***X* ^2^**	***df***	**Scaling correction factor**	**CFI**	**TLI**	**RMSEA**	**SRMR**	**CD**	**TRd**	**Δ *df***
Model 1	417.781	170	1.206	0.871	0.855	0.077	0.065	2.727	37.495	1 (*p* < 0.001)
Model 2	335.502	169	1.197	0.913	0.902	0.063	0.058			

Model 1, one-factor model; Model 2, two-factor model; CD, difference test scaling correction; TRd, Satorra-Bentler scaled chi-square difference.

### 3.3. Measurement invariance

We conducted configural measurement invariance by a multi-group model based on sex, and the model fit was acceptable [χ^2^(338) = 537.40, *p* < 0.001, CFI = 0.902, TLI = 0.890, RMSEA = 0.069, and SRMR = 0.068]. We further investigated metric invariance by constraining the factor loadings to be equal, and the model fit was reduced [χ^2^(356) = 565.823, *p* < 0.001, CFI = 0.897, TLI = 0.890, RMSEA = 0.069, SRMR = 0.085, ΔCFI = 0.005 (below the threshold of 0.010), and ΔTLI = 0.000 (below the threshold of 0.010)]. Finally, we investigated scalar measurement invariance by constraining the intercepts to be equal across sex, and it similarly yielded a reduced model fit [χ^2^(374) = 585.675, *p* < 0.001, CFI = 0.896, TLI = 0.894, RMSEA = 0.068, SRMR = 0.083, ΔCFI = 0.001 (below the threshold of 0.010), and ΔTLI = 0.004 (below the threshold of 0.010)]. Thus, configural, metric, and scalar invariance of C-FQ across sex were all supported.

### 3.4. Internal consistency reliability of C-FQ

We conducted an alpha reliability analysis to examine the internal consistency of emotional overinvolvement and criticism. Two subscales had excellent (>0.70) internal consistency, with Cronbach's alpha of 0.92 for both emotional overinvolvement and criticism.

### 3.5. Concurrent validity

Concurrent validity can be established with moderate-to-high correlations with other reliable instruments. We chose family function, quality of life, and caregiver burden as the benchmark in this study. Both dimensions of emotional overinvolvement and criticism are good predictors of the three chosen criteria. It is shown that the emotional overinvolvement dimension had a moderate negative correlation with family function [β = −0.25, 95% CI = (−0.21, −0.05), *p* = 0.001] and quality of life [β = −0.24, 95% CI = (−0.36, −0.07), *p* = 0.003], as well as a moderate positive correlation with care burden [β = 0.37, 95% CI = (0.40, 0.76), *p* < 0.001]. As for the criticism dimension, it had a high positive correlation with family function [β = 0.72, 95% CI = (0.28, 0.50), *p* < 0.001] and moderate positive correlation with care burden [β = 0.39, 95% CI = (0.41, 0.83), *p* < 0.001], as well as a moderate negative correlation with the quality of life [β = −0.23, 95% CI = (−0.36, −0.05), *p* = 0.008].

### 3.6. Associations of the C-FQ with socio-demographic and clinical characteristics

We examined the influence of socio-demographic and clinical characteristics on the two subscales of the C-FQ. Table 4 displays the statistical results. The results indicated that caregivers living with patients tended to score higher in EOI than those not living with patients. Additionally, EOI scores demonstrated significant differences in caregiving roles. The *post-hoc* test (the Dunn test) revealed that parents or spouses scored higher in EOI than siblings, but no significant difference existed between fathers, mothers, or spouses. Meanwhile, CC scores showed significant differences in education levels. The Dunn test revealed that caregivers with primary or college education reported higher CC scores than those with junior high school education. However, there were no significant differences between other education levels. Notably, contact time with patients per week showed a significant positive correlation with both EOI and CC scores. As for clinical characteristics, caregivers of patients taking medicine reported higher EOI scores than caregivers of patients not taking medicine. No significant differences were found concerning caregivers' age, gender, household monthly per capita income, and patients' illness duration.

**Table 4:** Univariate analysis of the association between C-FQ and socio-demographic characteristics.

**Caregivers' characteristics**	**EOI**			**CC**		
	***M*** **(*****SD*****)**	***z/*****H/*****r*** **(df)**	***p***	***M*** **(*****SD*****)**	***z/*****H/*****r*** **(df)**	***p***
**Age**	2.56 (0.47)	*r* (246) = *0.07*	0.252	2.41 (0.46)	*r* (246) = *−0.02*	0.734
**Gender**						
Male	2.57 (0.49)	*z* (246) *= −0.30*	0.767	2.42 (0.46)	*z* (246) *= −0.40*	0.689
Female	2.55 (0.46)			2.39 (0.47)		
**Educational level**						
Primary school	2.74 (0.37)	H (3) = 7.74	0.052	2.55 (0.50)	**H (3)** **=** **13.84**	**0.003**
Junior high school	2.50 (0.44)			2.31 (0.41)		
High school	2.56 (0.51)			2.43 (0.50)		
Undergraduate	2.63 (0.53)			2.58 (0.45)		
**Roles**						
Father	2.63 (0.48)	**H (4)** **=** **11.12**	**0.025**	2.44 (0.47)	H (4) = 5.56	0.234
Mother	2.60 (0.43)			2.41 (0.45)		
Spouse	2.54 (0.39)			2.42 (0.42)		
Siblings	2.26 (0.62)			2.22 (0.55)		
Others	2.44 (0.41)			2.46 (0.45)		
**Living with the patients**						
Yes	2.60 (0.45)	***z*** **(246)** ***=** **−2.82***	**0.005**	2.43 (0.45)	*z* (246) *= −1.68*	0.093
No	2.42 (0.53)			2.33 (0.49)		
Contact time with the patients per week (h)	2.56 (0.47)	***r*** **(246)** **=** ***0.27***	**< 0.001**	2.41 (0.46)	***r*** **(246)** **=** ***0.17***	**0.009**
**Household monthly per capita income (CNY)**						
Under 3,000	2.64 (0.53)	H (3) = 5.39	0.145	2.46 (0.50)	H (3) = 5.37	0.147
3,001–5,000	2.58 (0.45)			2.42 (0.48)		
5,001–10,000	2.43 (0.50)			2.33 (0.36)		
More than 10,000	2.68 (0.19)			2.45 (0.39)		
**Illness duration of patients**	2.56 (0.47)	*r* (246) = *−0.09*	0.155	2.41 (0.46)	*r* (246) = *−0.07*	0.303
**Taking medicine (patients)**						
Yes	2.58 (0.47)	***z*** **(246)** ***=** **−2.14***	**0.032**	2.41 (0.46)	*z* (246) *= −0.72*	0.469
No	2.37 (0.49)			2.35 (0.54)		

Statistically significant differences at *p* < 0.05, based on Mann–Whitney U-test for two independent samples, Kruskal–Wallis one-way analysis of variance by ranks, and Spearman's rho correlation coefficient. EOI stands for emotional overinvolvement; CC stands for criticism.

## 4. Discussion

The primary aim of this study was to adapt the FQ into the Chinese version and evaluate its psychometric properties in a sample of family relatives of people with schizophrenia. Expressed emotion has been widely studied in many cultural contexts. However, there were not many empirical results from the Chinese sample. The lack of appropriate measurements could hinder studying expressed emotion in China. Thus, as a time- and cost-effective instrument, the C-FQ would be beneficial for research about expressed emotion and clinical practice based on expressed emotion theory in China, as well as the cross-cultural comparison of expressed emotion theory between the Chinese context and other cultures. Overall, the C-FQ presents good psychometric properties, including good structure validity, reliability, and concurrent validity.

The original FQ displayed a two-factor structure: emotional overinvolvement and criticism. Our CFA results indicated the same two-factor structure of the C-FQ. Specifically, the one-factor model showed a poor model fit to the data (CFI and TLI < 0.9), while the two-factor model showed a good model fit (CFI and TLI > 0.9). Using Satorra-Bentler scaled chi-square difference test to compare the fitness of these two models, we found that the fit of the two-factor model was significantly better than the one-factor model. This two-factor solution is in line with the factor structure of other non-English FQ versions (i.e., Spanish, Italian, and Brazilian Portuguese). Based on that, it would be essential to distinguish different aspects of the emotional experience of family members of people with mental illness.

Generally, C-FQ items showed good factor loadings in the two-factor model. However, item 17 showed a low factor loading (0.37), while the loadings of other items ranged from 0.56 to 0.78. The low factor loading of item 17 may be due to its contents (“He/she is an important part of my life”). Most caregivers are close family members of people with schizophrenia, such as fathers, mothers, and spouses. Thus, a feeling that sons/daughters/spouses are an essential part of parents'/spouses' life could be an expected condition among them. Considering that this feeling is common and natural among close family members, it cannot reflect the overinvolvement of emotions. In addition, it may be unable to discriminate between family relationships with and without people with mental illness. The low factor loading of item 17 was also found in other translated versions of the FQ, including the Greek version [loading is 0.42; (36)] and the Spanish version [0.14 in a sample of mothers, 0.22 in a sample of fathers; (38)]. The Italian version deleted item 17 because of its extremely low value (0.16) of item-total correlation and non-significant loading (35). Given that, item 17 may need an amendment to achieve better measurement validity. To modify it as “He/she is the *most* important part of my life” might be helpful.

In this study, we further test the stability of the C-FQ's factorial structure across sex by the analysis of measurement invariance. Our results showed that the configural, metric, and scalar invariance of C-FQ across sex were all supported, which indicated that male and female caregivers had the same C-FQ factor structure and interpretation of the scale items. As far as we know, this is the first study to examine the measurement invariance of the FQ across sex. In previous studies, caregivers' levels of emotion are compared without testing the factorial structure consistency of the measurement [e.g., (35, 38)]. A different examination of sex, however, would be meaningless if the scale did not measure the latent construct equally for men and women. Thus, our findings gave evidence to support the examination of sex differences in expressed emotion. We also conducted an alpha reliability analysis to examine the internal consistency of the two C-FQ subscales. The results indicate that emotional overinvolvement and criticism showed excellent reliability indexes with Cronbach's α coefficient.

Finally, we examined the concurrent validity of C-FQ by testing its ability to predict constructs related to caregiving experience, family function, and wellbeing. The two subscales are both effective predictors of care burden, family function, and caregiver's quality of life. Specifically, caregivers who are over concerned with the patient or show a higher level of criticism toward the patient tend to experience a higher level of care burden, poorer quality of life, and poor family function. These results are in line with existing research on expressed emotion across different cultures (4, 6, 10). The good concurrent validity of C-FQ suggests that expressed emotion in Chinese contexts can be well-measured with a self-reported method.

Despite the interesting findings of our study, several limitations should be noted. First, we did not examine the test–retest reliability of this instrument. Many family intervention studies would examine changes in expressed emotion before and after the intervention. Therefore, a deep exploration of the test–retest reliability of the C-FQ would help researchers decide whether to use it for measuring intervention effects. Second, we only successfully recruited caregivers of people with schizophrenia to participate in this study. Future research could examine the psychometric properties of the C-FQ by collecting data from different clinical samples (e.g., depression, eating disorders, and dementia) to examine its generalizability. Third, we are unable to validate the cutoff scores of the C-FQ to distinguish high and low levels of expressed emotion. This is limited by the lack of trained psychiatrists to conduct the Camberwell Family Inventory (CFI) for comparison. Future studies could validate the cutoff scores of expressed emotion in the Chinese context by using the CFI for comparison and taking possible cultural differences into account. In addition, future research should also (1) collect data on symptoms and rehospitalization to test the C-FQ's predictive power in clinical outcomes and relapse of patients; (2) further evaluate C-FQ psychometric properties with diverse samples of caregivers from different socio-cultural backgrounds; and (3) consider developing a valid patient report version of C-FQ that enables researchers to explore the interaction between patients and their caregivers using dyadic data (i.e., the EE caregivers expressed and the EE the patients perceived). Finally, although the self-report measure is easy to administer and less time-consuming, its methodological disadvantages should also be acknowledged. Self-report data may be affected by social desirability. Given the negative connotation of the high-EE construct, most FQ items are negatively worded. Participants might be reluctant to give a strong agreement response to those items due to the impact of social desirability. Therefore, short self-report measures are best viewed as probing for high-EE attitudes rather than a replacement for the full CFI.

## 5. Conclusion

Overall, the Chinese version of the Family Questionnaire is a reliable and valid measurement to assess the expressed emotion of caregivers of people with schizophrenia in the Chinese context. The C-FQ presents good reliability, construct validity, and concurrent validity. Our results also indicated that C-FQ has the same factor structure across sex, which supports the future researcher to compare the expressed emotion level among male and female groups in the Chinese context. Despite its short length, the C-FQ effectively measures two core elements of EE and requires no special training for the administrator. Thus, given the time constraints and shortage of training resources in Chinese clinical settings, it could be a preferred tool. A simple and short measure would also reduce respondents' burden, thereby increasing their willingness to respond. The C-FQ results can inform healthcare professionals about EE levels in family members in terms of critical comments and emotional overinvolvement. By obtaining clinical information on these aspects, healthcare professionals can develop timely and tailored interventions to improve the family environment of patients. The intervention on caregivers' EE levels may also help patients combat the negative impact of self-stigma. Evidence indicates that caregivers' high-EE levels may contribute to patients' self-stigmatization (79, 80). The reduction of caregivers' EE levels may keep people with mental health disorders from internalizing stigma attitudes and allow them to live more fulfilling lives. Except for clinical implications, the C-FQ can be used to advance our understanding of how EE manifests in China and how it affects patients and their caregivers through empirical research. In turn, these empirical studies would benefit the cross-cultural investigation of EE.

## Data availability statement

The raw data supporting the conclusions of this article will be made available by the authors, without undue reservation.

## Ethics statement

The studies involving human participants were reviewed and approved by the East China Normal University Committee on Human Research Protection (IRB No: HR 012-2019). The participants provided their written informed consent to participate in this study.

## Author contributions

YP was responsible for formulating research aims and designs, data collection, data analysis, and draft writing and revising. EX was involved in data analysis and original draft writing. YL and LS were responsible for the data collection. JX supervised this study and made critical revisions to the study. All authors contributed to the article and approved the submitted version.
