# Supplementary material for: Psychometric properties of the Chinese version of the Family Questionnaire among the caregivers of people with schizophrenia
Source: Front Public Health. 2023 Jul 14;11:1200130. doi: 10.3389/fpubh.2023.1200130 (PMC10377673; doi:10.3389/fpubh.2023.1200130)
Supplement: Supplementary file 1 [file Table_1.DOCX]

Supplementary Table 1. The original English and Chinese versions of the Family Questionnaire

| English version | Chinese version |
| --- | --- |
| 1 I tend to neglect myself because of him/her. | 1因为他/她，我往往忽略了自己。 |
| 2 I have to keep asking him/her to do things. | 2我必须一遍遍地要求他/她做某些事情。 |
| 3 I often think about what is to become of him/her. | 3我经常会想他/她将来该怎么办。 |
| 4 He/she irritates me. | 4他/她会惹我生气。 |
| 5 I keep thinking about the reasons for his/her illness. | 5我总是会想他/她得这个病的原因。 |
| 6 I have to try not to criticize him/her. | 6我需要控制自己尽量不去责怪他/她。 |
| 7 I can’t sleep because of him/her. | 7因为他/她，我无法入睡。 |
| 8 It’s hard for us to agree on things. | 8在许多事情上，我们的意见很难达成一致。 |
| 9 When something about him/her bothers me, I keep it to myself. | 9当与他/她有关的事情困扰我时，我会把它埋在心里。 |
| 10 He/she does not appreciate what I do for him/her. | 10他/她并不感激我为他/她所做的一切。 |
| 11 I regard my own needs as less important. | 11和他/她的需求相比，我认为我自己的需求不那么重要。 |
| 12 He/she sometimes gets on my nerves. | 12他/她有时候让我心烦意乱。 |
| 13 I’m very worried about him/her. | 13我非常担心他/她。 |
| 14 He/she does some things out of spite. | 14他/她会出于恶意做一些事情。 |
| 15 I thought I would become ill myself. | 15我觉得我自己可能也会生病。 |
| 16 When he/she constantly wants something from me, it annoys me. | 16当他/她不断地想从我这得到什么的时候，我会感到很烦躁。 |
| 17 He/she is an important part of my life. | 17他/她是我生命中重要的一部分。 |
| 18 I have to insist that he/she behave differently. | 18我不得不要求他/她在行为举止上做出改变。 |
| 19 I have given up important things in order to be able to help him/her. | 19为了能帮助他/她，我放弃了一些重要的东西。 |
| 20 I’m often angry with him/her. | 20我经常生他/她的气。 |

*Note*. The odd-numbered items belong to the dimension of emotional overinvolvement, and the even-numbered items belong to the dimension of criticism.
